# Key populations and human rights in the context of HIV services rendition in Ghana

**DOI:** 10.1186/s12914-017-0129-z

**Published:** 2017-08-02

**Authors:** Amos Laar, Debra DeBruin

**Affiliations:** 1 Department of Population, Family, and Reproductive Health, School of Public Health, College of Health Sciences, University of Ghana, Box LG 13, Legon, Accra Ghana; 2 Center for Bioethics, University of Minnesota, 410 Church Street S.E MN, Minneapolis, 55455-0346 USA

**Keywords:** Health rights, Positive rights, Negative rights, HIV, Key populations, Ghana

## Abstract

**Background:**

In line with its half century old penal code, Ghana currently criminalizes and penalizes behaviors of some key populations – populations deemed to be at higher risk of acquiring or transmitting Human Immunodeficiency Virus (HIV). Men who have sex with men (MSM), and sex workers (SWs) fit into this categorization. This paper provides an analysis of how enactment and implementation of rights-limiting laws not only limit rights, but also amplify risk and vulnerability to HIV in key and general populations. The paper derives from a project that assessed the ethics sensitivity of key documents guiding Ghana’s response to its HIV epidemic. Assessment was guided by leading frameworks from public health ethics, and relevant articles from the international bill of rights.

**Discussion:**

Ghana’s response to her HIV epidemic does not adequately address the rights and needs of key populations. Even though the national response has achieved some public health successes, palpable efforts to address rights issues remain nascent. Ghana’s guiding documents for HIV response include no advocacy for decriminalization, depenalization or harm reduction approaches for these key populations. The impact of rights-restricting codes on the nation’s HIV epidemic is real: criminalization impedes key populations’ access to HIV prevention and treatment services. Given that they are bridging populations, whatever affects the Ghanaian key populations directly, affects the general population indirectly.

**Summary:**

The right to the highest attainable standard of health, without qualification, is generally acknowledged as a fundamental human right. Unfortunately, this right currently eludes the Ghanaian SW and MSM. The paper endorses decriminalization as a means of promoting this right. In the face of opposition to decriminalization, the paper proposes specific harm reduction strategies as approaches to promote health and uplift the diminished rights of key populations. Thus the authors call on Ghana to remove impediments to public health services provision to these populations. Doing so will require political will and sufficient planning toward prioritizing HIV prevention, care and treatment programming for key populations.

## Background

In addressing the multitude of health and human rights issues globally, ethicists, public health experts and human rights scholars have developed various frameworks. Frameworks for analyzing public health programs [1], public health practice [2] health policy reforms [3], and human rights impact assessment of public health policies [4] exist. Marieke *ten* Have et al. thoroughly examined these and other frameworks [5]. Human rights scholars have also argued for both national and international responsibility pursuant to the human rights to health enshrined in the Constitution of the World Health Organization [6].

One polemical health and human rights issue that has attracted emotive national discourse in Ghana is whether or not persons engaging in legally prohibited behaviors have rights. Rightly referred to as key populations^1^ and including men who have sex with men (MSM), persons who inject drugs (PWID), transgender persons, prisoners, and sex workers (SWs), they are key to the epidemic’s dynamics and key to its response. This paper focuses on two of these populations: MSM and SWs. Generally considered deviants in Ghanaian culture, these groups are at the receiving end of restrictive and arguably rights-restricting penal codes [7–9]. Even though many calls exist for decriminalization of these groups [7, 10–12], Ghana currently criminalizes and penalizes same sex relationships, solicitation for sex, and sex work [13].

Several authors have written about laws criminalizing same-sex sexual conduct, leading to arbitrary arrests and detentions. They note that these laws are used to threaten arrest and punish individuals for engaging in same-sex sexual conduct and sex work [9, 12, 14]. Punitive provisions criminalizing homosexuality and sex work not only weaken human rights protections [15] but also limit the efforts of public health personnel to reach these populations with Human Immunodeficiency Virus (HIV) and other health interventions [16]. Of note, on several occasions public advocacy on behalf of MSM rights has contributed to a backlash or re-enforcement of negative measures; including more rights-limiting bills in Uganda and Nigeria [17, 18], and public outcry in Ghana and Senegal [11, 16]. The Ghana office of UNAIDS attempted intervening in a number of ways. UNAIDS worked with other stakeholders toward removing punitive laws, policies, and practices, that block effective responses to AIDS in Ghana. Such included development of a code of conduct for the Ghana Police which focused on punishment for members who infringed on the Human Rights of MSM and Female Sex Workers (FSWs). Female Sex Workers in four of ten regions of Ghana were trained as focal points. Legal services were also procured for four MSM who were prosecuted on allegations of “Unnatural Carnal Knowledge” [11].

There is a clear trend toward an expansion of litigation opportunities in all regions of the world as individuals and non-governmental organizations (NGOs) seek to hold governments accountable for public health obligations [19]. The link between rights, health policies, and public health outcomes has received little attention in Ghana (particularly empirical research and policy analysis). To this end, there is a pressing research and advocacy need for the local health and human rights community to clarify the connections between human rights and public health.

To date, quite a lot is known about HIV and Ghana’s response to HIV. Awareness about HIV is near universal. Epidemiologically, Ghana is classified as having a generalised^2^ HIV epidemic. According to the latest nationally representative population-based survey, HIV prevalence in Ghana was 2.0% in 2014, having decreased marginally from a high 3.2% in 2006 [20]. However, the 2016 HIV Sentinel Survey reports a national HIV prevalence of 2.4%, a second consecutive increase from 1.6% in 2014 and 1.8% in 2015. According to the latest modes of transmission study (MoT), the majority of new HIV infections (72.3%) is occurring among the general population, but regular partners of FSWs together account for nearly one-quarter (23.0%) of new HIV infections. Sex work accounted for 18.4% of all new infections in 2014 having declined from 27% in 2009. The report attributes the decline to declines in new HIV infection among the following sub-groups: clients of female sex workers (from 14.7% in 2009 to 5.0% in 2014); sex workers (from 5.4% in 2009 to 2.9% in 2014) [20]; female partners of clients of sex workers (from 19% in 2009 to 10.4% in 2014). The estimated size of MSM and FSWs in Ghana are respectively 30,600 and 52,000 (IBBSS 2011). Close to 18% of MSM are living with HIV – contributing 3.6% of new HIV infections. Even though HIV prevalence among FSWs has been decreasing consistently over the last 15 years, it is still unacceptably high. Prevalence among female sex workers in Accra decreased from 50% in 2001 to 11% in 2011.

Despite restrictive laws, key populations continue to receive HIV testing without serious hindrances. Barriers to accessing AIDS care include: (1) transport costs (distance) to the limited number of ART sites; (2) preponderance of standalone ART clinics that facilitates and sustains stigma because anyone stepping into these clinics is assumed to be HIV+; (3) discriminatory attitudes of some health workers and law enforcement agencies toward key populations [9].

Even though Ghana has managed to create universal awareness about HIV, significant challenges and gaps remain. Such challenges include the lack of data on a group known to be relevant to the dynamics of the epidemic. For instance, personal anecdotes hold that Ghana’s restrictive laws serve as barriers to providing HIV care, and receipt of same by key populations. Such laws are briefly are outlined herein. Under the 1960 Ghanaian Criminal Code, same-sex sexual conduct is a criminal offence. Subsection (1)(b) of Section 104 of Ghana’s Criminal Code criminalizes consensual “unnatural carnal knowledge,” where “unnatural carnal knowledge^3^” is interpreted (rightfully or wrongfully) to include same-sex sexual conduct. Section 274 of the Criminal Offences Act 1960 (Act 29) criminalizes the act of prostitution. It states that “any person who knowingly lives wholly or in part on the earnings of prostitution; or is proved to have, for the purposes of gain, exercised control, direction or influence over the movements of a prostitute in such manner as to aid, abet or compel the prostitution with any person or generally, shall be guilty of a misdemeanour.” Additionally, Section 275 of the same Act also states that “any person who in any public place persistently solicits or importunes to obtain clients for any prostitute or for any other immoral purpose shall be guilty of a misdemeanor”. To address these and other barriers, Ghana’s response to HIV seeks to utilize trained health facility nurses as case managers for HIV-positive key populations. Planning for assessments of human rights issues and the quality of service for key populations is underway.

This paper discusses rights; it sketches the evolving interaction between rights violations and HIV risk. It specifically addresses the rights of key populations (MSM, SWs) to public health services, and considers whether current legal actions against key populations by state actors violate their rights to health. The paper then discusses the promise of two approaches – decriminalization, and a more instrumentalist harm reduction approach -- in ensuring accountability for country level public health actions. The paper implores both scholars and practitioners to conduct more empirical research and policy analysis on the subject.

## Relevant international human rights norms in the context of HIV and key populations

Although no global human rights treaty expressly addresses HIV [21], there are a wealth of norms and principles in general human rights treaties that are relevant to HIV and to the protection of persons affected by the epidemic, including key populations. The international legal frameworks developed since the founding of the United Nations (UN) identify individual rights-holders and their entitlements, together with corresponding duty-bearers and their obligations. Human rights are protected under international law, under regional systems, and by national constitutions [22]. Some of the basic human rights are asserted in the morally binding Universal Declaration of Human Rights (UDHR), adopted in 1948 by the General Assembly of the United Nations. Ghana is a signatory. Founded upon the non-derogable right to life, the UDHR affirms in Article 25/1 “everyone has the right to a standard of living adequate for the health and wellbeing of himself…” [23]. Subsequent international human rights instruments have not only expanded this, but have also made human rights law legally binding. The International Covenant on Civil and Political Rights (ICCPR) [24] requires member states to respect and ensure civil and political rights. Codified in the International Covenant on Economic, Social and Cultural Rights (ICESCR), and in line with the WHO Constitution. Article 12 of ICESCR states that “the States Parties recognize the right of everyone to the enjoyment of the highest attainable standard of physical and mental health” [25]. Building from these standards, a wide range of UN agencies and advocacy groups have increasingly invoked a “human rights-based approach” to health as a means to frame the public health policy environment and to facilitate government accountability [26].

In his comprehensive human rights analysis of HIV-specific legislation in sub-Saharan Africa, Eba [21] refers to these norms, noting that the open-ended grounds for prohibiting discrimination based on ‘other status’ in the ICCPR and the ICESCR can be interpreted to include non-discrimination based on health and HIV status. Thus, the provisions in these global treaties relating to the rights to liberty, security, equality, health, education, free and fair trial, among others, are also relevant to the HIV epidemic and for persons affected by HIV including key populations [27].

With the emergence of HIV treatment as a means to prolong life, human rights advocacy broadened its emphasis on the prevention of rights violations relating to discrimination and stigmatization to include a focus on the provision of antiretroviral drugs, within the broad theme of treatment access [19]**.** Currently empowered individuals and nongovernmental organizations raise human rights arguments and claims, pressing their governments to deliver medications as an immediate matter of life and death**.** Not all such claims are honored.

## Criminalizing the behaviors of key populations: a bird’s eye view

In many countries of the world, various rights-restricting policies and criminal laws exist in relation to the activities of MSM, SW and other key populations [8]. These laws and policies criminalize activities of key populations and, as noted below, also at times penalize others for offering supportive services to these groups or simply failing to report them to authorities [28]. Amon et al. write about the use of antiquated and non-specific legal codes to harass, intimidate or justify the use of force against sex workers [29]. A 2009 Joint United Nations Programme on HIV/AIDS (UNAIDS) guidance note on HIV and sex work cited the existence of specific discriminatory laws against homosexuality and transgenderism [10]. Overs and Hawkins cite a Malawian Newspaper decrying the notorious use of vagrancy laws to criminalize sex workers in Malawi [7]. Overs and Hawkins also argue that in settings where these groups are not directly criminalized, rights-neutral policies result in widespread stigmatization and discrimination with impunity. For instance, the 2012 ‘country progress report’ by the Ghana AIDS Commission (GAC) to the UNAIDS notes that key populations have difficulties accessing HIV prevention services not necessarily due to fear of being arrested, but as a result of stigma and social hostility [30]. Thus purported neutrality undermines the right to health.

An analysis by Persson et al. documented that consensual same-sex sexual activity is illegal in approximately 76 to 86 countries globally [8]. Thirty eight of the 54 African countries criminalize same-sex relationships and punishment ranges from imprisonment to death [31]. Blackmail and extortion of this group are on the rise [32]. Viewed as Euro-American decadency, according to some scholars, same-sex relationships are feared, despised and regarded with disdain and disgust in certain African communities [31]. Some see such relationships as a condition that ought to be cut out and exposed before it becomes a “spreading cancer” in other men. Evidence from Senegal, Malawi, and Uganda support this. In Senegal, for example, leaders and staff of a program developed to support MSM were sentenced to 5 years for sodomy [28]. Similar actions have occurred in Malawi, and Uganda. The infamous Ugandan Anti-Homosexuality Bill (once referred to as the “Kill the Gays Bill”) received global criticism in times past [17]. It was proposed in the Bill that sodomy be made a capital offense, and that failure to report individuals suspected of engaging in homosexual behaviors is criminal [28].

Djomand et al. [33] discuss, among others, the legal and human rights challenges related to the HIV epidemic among key populations in West Africa. Sex work is illegal in most West African countries with numerous punitive legal approaches, though these are unevenly enforced [14, 33]. Senegal is the lone exception, where prostitution has been legal and regulated since 1969 [34]. Likewise, MSM are highly stigmatized as same-sex intercourse is criminalized across West Africa and may be punishable by lengthy prison terms or death [35, 36]. A recent Nigerian law mandates a 14-year prison sentence for anyone entering a same-sex union and a 10-year term for ‘a person or group of persons who supports the registration, operation and sustenance of gay clubs, societies, organizations, processions or meetings [18]. In some countries, healthcare providers are pressured by governmental authorities to disclose the identities of their MSM clients, rendering this political environment a deterrent for seeking care and/or treatment services [36].

A related key populations policy analysis covering Côte d’Ivoire, Ghana, Togo, Benin, and Nigeria and Burkina Faso (all West African countries) show that all countries in the analysis except Benin have at least one law that criminalizes behaviors of one or more key populations; in addition, soliciting for sex work is illegal in four countries; sex work itself is only criminalized in Ghana and Nigeria [14] (see Table 1). Non-custodial alternatives to prison are available only in Ghana and Togo. Same-sex sexual behavior is criminalized in Ghana, Togo, and Nigeria, with non-custodial alternatives to prison unavailable in any of these countries. Nigeria has the harshest penalties, while Côte d’Ivoire and Burkina Faso have no laws regarding same-sex sexual behavior. Only three countries (Côte d’Ivoire, Ghana, and Togo) had policies protecting NGOs and service providers from prosecution on charges of aiding and abetting (see Table 1 below).

**Table 1:** Existence of criminalization laws and policies in Abidjan-Lagos Corridor Countries and Burkina Faso

Policy	Abidjan-Lagos Corridor Countries					Other WA
	Côte d’Ivoire	Ghana	Togo	Benin	Nigeria	Burkina Faso
1. Sex work is not criminalized	✓	X	✓	✓	X	✓
2. Soliciting for sex work is not criminalized	X	X	**X**	✓	X	X
3. Same-sex sexual behavior is not criminalized	✓	X	X	✓	X	✓
4. Transgender identity or gender nonconformity is not criminalized	✓	✓	✓	✓	✓	✓
5. Alternatives to prison are available for people convicted offenses related to sex work, prostitution, or solicitation	X	✓	✓	N/A	X	X
6. Alternatives to prison are available for people convicted to offences related to same-sex sexual behavior	X	X	X	N/A	X	N/A
7. NGOs and providers are explicitly protected from prosecution charges of aiding and abetting	✓	✓	✓	X	X	X

Source: Duvall et al. [14]

X = does not affirm statement in the first column e.g. Statement 2: With the exception of Benin, soliciting for sex is criminalized in all the other five countries

✓ = affirms statement in the first column e.g. Statement 4: In all six countries, there is no existing of a law criminalizing gender noncomformity

## Criminalizing the behaviors of MSM and SW in Ghana

The world over, legal and policy frameworks have impact on persons living with HIV (PLHIV) and key populations [37]. Punitive provision for HIV transmission and criminalization of homosexuality and sex work can also weaken human rights protections [15]. In Ghana, relevant laws and policies affecting key populations exist. Subsection (1)(b) of Section 104 of Ghana’s Criminal Code criminalizes consensual “unnatural carnal knowledge.” Chapter 6 Section 104 of Ghana’s Criminal Code states:

(1) Whoever has unnatural carnal knowledge

- of any person of the age of sixteen years or over without his consent shall be guilty of a first degree felony and shall be liable on conviction to imprisonment for a term of not less than five years and not more than twenty-five years; or
- of any person of sixteen years or over with his consent is guilty of a misdemeanor; or
- of any animal is guilty of a misdemeanor.

Because “unnatural carnal knowledge” is used to refer to same-sex sexual conduct, This law is used to threaten, arrest and punish individuals for engaging in same-sex sexual conduct [12] Also, section 274 of the Criminal Offences Act 1960 (Act 29) criminalizes the act of prostitution. It states

“…any person who knowingly lives wholly or in part on the earnings of prostitution; or is proved to have, for the purposes of gain, exercised control, direction or influence over the movements of a prostitute in such manner as to aid, abet or compel the prostitution with any person or generally, shall be guilty of a misdemeanour.”

Additionally, Section 275 of the same Act states that “any person who in any public place persistently solicits or importunes to obtain clients for any prostitute or for any other immoral purpose shall be guilty of a misdemeanor”.

As noted above, international human rights norms emphasize the prevention of rights violations relating to discrimination and stigmatization as well as focus on the access to HIV preventive services and treatment. While no specific legislation outlaws HIV-related discrimination in Ghana, the country’s legal frame work does provide broad human rights protections for PLHIV. The 1992 Constitution of the Republic of Ghana protects all persons *(including PLHIV and key populations; emphasis is ours)* against discrimination and upholds fundamental human rights. It states: “All persons shall be equal before the law. A person shall not be discriminated against on grounds of gender, race, ethnic origin, religion, creed or social economic status” [38]. Of note, sexual orientation is not listed among the protected statuses. The Patient Charter, the Ghana AIDS Commission Act of 2002 (Act 613), the HIV and AIDS Bill, and the National HIV policy of 2013 are all rights upholding.

Health-specific policies that do not carry the force of law can also provide a basis for protecting human rights. A Patient’s Charter (a morally-binding service provision guidance document that outlines the obligations and responsibilities of healthcare provider and service user/client) protects patients in the Ghanaian public health system from discrimination based on culture, ethnicity, language, religion, gender, age, type of illness, or disability. Through the prohibition of discrimination by type of illness, the Patient’s Charter forbids HIV-related discrimination. Unfortunately such documents do not wield the same level of legal force as the Criminal Code of the Republic of Ghana, 1960 [13]. Although the Government of Ghana (GoG) favors a legal framework, free from discrimination, that supports political, economic, social and cultural responses, together with other strategies to address HIV, human rights violations and related stigma and discrimination of persons infected with and affected by HIV is pernicious and pervasive [39]. Human rights of PLHIV and key populations most affected by HIV are often violated, with negative implications for health outcomes [12]. The prevailing view of MSM is that it is a Euro-American perversion which, if not confronted, can contaminate other minds. Such a framing poses serious challenges to attempts to provide services to this group. In Ghana for instance, staff of non-governmental organizations (NGOs) providing services to key populations in nontraditional spaces face the real possibility of being arrested in periodic Police swoops. In 2016 alone, such swoops targeting sex workers led to the arrest of 29 on July 5 2016 [40], 18 on March 1 2016 [41], and 19 on February 29 2016 [42]. The public health impact of criminalizing behaviors of key populations is significant. According to a local study [43], key determinants of HIV in Ghana include marginalization of key populations, multiple concurrent partnerships, and stigma and discrimination. The 2012 ‘country progress report’ by the Ghana AIDS Commission to UNAIDS notes that key populations have difficulties accessing HIV prevention services due to stigma and discrimination, social hostility, fear of losing jobs and families, and even verbal and physical violence [30].

Trend analysis of HIV sero-prevalence among key populations is not possible due to lack of data. Due to such lack of data, some stakeholders have argued that there are no systematic human rights constraints to services, there are various barriers that can exist and may deter key populations from seeking testing and ART. For instance, in 2016, 21 of 78 reports by PLHIV and key populations to the Commission for Human Rights and Administrative Justice (CHRAJ) were complaints about disclosure of confidential health information compared to three reports of denial of health services. The CHRAJ report does not provide additional context but discussions with key populations during the Country Dialogue suggest such disclosures of personal health information are often made by one key populations to another and discourages key populations from any activity that may expose their HIV status to their peers. The authors hold the view that such absence of data is not only an ethics issue, but is also an issue of deep public health concern and a human rights issue that ought to be addressed. In an era where healthcare policies, programming, planning, service provision are data-driven, lack of strategic information impedes service provision and uptake.

## Upholding the rights of key populations

This paper argues that key populations’ rights to public health services should be respected. The emerging rubric of theoretical human rights analysis anchors the reasoning in this paper. Theoretical human rights analysis, Benjamin Mason Meier notes, has become a principal strategy for realizing international treaty obligations for the human right to health – providing causes of action for the public’s health and empowering individuals to raise human rights claims for care (in the current context HIV prevention, treatment, and care) [19]. Over the past two decades, several advocates, mostly from the developed world, and in a few instances from southern Africa, have laid the groundwork on the marriage of human rights and HIV policy. The work of Robins illustrates the translation of human rights from principle to practice in the global response to HIV [44]. In particular, the Treatment Action Campaign (TAC), a South African AIDS activist organization, has been credited with forcing the reluctant government of former South African President Thabo Mbeki to begin making antiretroviral drugs available to South Africans. As Meier et al. put it, TAC’s work transformed aspirational declarations into justiciable obligations; currently, human rights actions now run through the veins of many national HIV policies and programs [19].

Various policies in Ghana – including the actions of Act 29 of the 1960 Criminal Code of Ghana and inactions of the NSP and national HIV policy -- directly and indirectly prevent or inhibit key populations’ access to services – by extension a curtailment of their right to health. In 2001, all UN member states including Ghana endorsed a commitment to protect human rights in the global fight against HIV and to ensure universal access to HIV prevention, treatment, care, and support [45]. Yet efforts at pressing for key populations’ right to health (which includes universal access to HIV prevention services) have been ineffective in compelling nation states to fulfill this promise. A popular argument in support of limiting the rights of key populations has been the protection of public morals.

Invoking the above rights arguments and doctrine, the UN Human Rights Council in 2009 adopted a resolution that urged states to eliminate laws that are counterproductive to HIV prevention, treatment and care including those that violate the rights of populations key to the dynamics of the epidemic and particularly affected by it [10]. The UNAIDS Joint Outcome Framework of the same year made the removal of laws, policies and practices that block effective action on HIV a priority. It also mentioned sex work as part of a broader human rights agenda [46]. Other international organizations in their bid to address the situation for MSM have launched other programs. The United Nations Educational, Scientific, and Cultural Organization (UNESCO), for example, has been supportive of efforts that call on governments to eliminate the unacceptable and devastating prevalence of lesbian, gay, bisexual, transgender and intersex bullying around the world [47].

As established earlier, Ghana is a signatory to various global human rights instruments including the commitment pledging to “enact, strengthen or enforce, as appropriate, legislation, regulations and other measures to eliminate all forms of discriminatory tendencies, and to ensure the full enjoyment of all human rights and fundamental freedoms of vulnerable population”. Having presented the human rights declarations and conventions to which Ghana is a signatory, we discuss the significance of the moral vision encompassed in them and the moral/legal significance of the steps that Ghana has taken with respect to them. This paper will now address approaches that Ghana can take to better fulfill this commitment in its HIV response.

## Key populations and harm reduction

There is now broad agreement that harm reduction and human rights share common cause, each reflecting core principles of the other [48]. The Commentary on ICESCR (General Comment 14, 2000) argues that the right to health includes the right to seek, to receive, and to impart information. A harm reduction approach captures policies, programs, and practices that seek to reduce harms associated with an activity without requiring prohibition of the activity, for example, needle exchange programs and condom distribution. Gruskin’s work expounds on the conceptual linkages of harm reduction approaches to human rights. Human rights provides normative validation for harm reduction, namely the legal obligation to act on the evidence of effective interventions to reduce harm and thus protect rights, harm reduction offers evidence of the effectiveness of human rights-based approaches to health [49]. Further, Erdman concurs with others that international human rights law has evolved to the point where it now imposes. . . obligations on governments to provide, and to refrain from interfering with life-saving goods of their citizenry (see Erdman, 2011; pp. 4) [50]. A review of the literature on harm reduction and human rights demonstrates that some ground has been lost and opportunities missed by the failure of some public health professionals to exploit harm reduction in the discourses of key populations’ criminalization. This paper pays heed. It argues that harm reduction approaches in HIV response in Ghana would promote the human rights of MSM and SWs.

The neutrality principle of harm reduction calls for a non-judgmental approach to the underlying activity. Harm reduction concerns only the risks and health-related harms of an activity, not whether the activity is normatively right or wrong. In this context, sex work, or same sex relationships, are thus not the problem to be solved. Rather, harm reduction programs seek to address the health risks relating to these behaviors (and the negative impacts of a public health systems’ failure to meet the health needs of these populations). These impacts include increased risk of HIV infection, debility, disability, and death of key population as well as the general population. In the context of HIV programming, key populations are not only ‘criminals’, but are also contributors to increased HIV risk in both key populations and the general population.

Evidence show that criminalizing the activities of key populations hinders provision of HIV services to them and is thus self-defeating. Jurgens et al. speak to the fact that criminalization limits the ability of healthcare workers to provide essential HIV prevention services [28]. Wade et al. show in Senegal an HIV prevalence of 21.5% among MSM compared to 0.2% among other men [51]. Local estimates demonstrate the prevalence of HIV among key populations in Ghana to be over ten-fold higher than in the general population in Ghana [52]. With these disparities in mind, Persson et al. describe criminalization of key populations as “adding insult to injury” [8]. Acknowledged as a bridging population in Ghana (because the Ghanaian key populations sexually interact with the general population), there is no denying the fact that whatever affects such MSM/SWs directly affects the general population indirectly. In this context, criminalization of their status and actions are medically counterproductive to national gains in curbing the menace of the HIV disease, not only in key populations, but in the country overall.

Further, harm reduction approaches humanistically extend the neutral reservation of judgment beyond the activity to the individuals who engage in it. Regardless of imputed moral status or deviance from legal norms, all individuals should be treated with respect and deserving of concern for their health and lives. Thus, harm reduction approaches embrace humanism, an “acceptance of the simple humanity of the drug user (*or MSM, or FSWs; emphasis in parenthesis is ours*), her connection to the rest of us.” [50]. The value-neutrality of a health discourse is explicitly used in support of the humanistic commitment. Whatever objectives underlie laws criminalizing the behaviors of key populations, the state should not require the sacrifice of health or life to achieve them. To do so would be inhumane and degrading. The humanistic commitment of harm reduction applies not only to the entitlement to healthcare, but also to the manner in which it is provided: According to General Comment #14 (2000), health services, per international human rights law, are required to be “acceptable.” This means services are respectful, ensure free and informed decision-making, guarantee confidentiality, and are informed by the needs and perspectives of the individual.

Although largely adopted in criminal legal frameworks (concerned with abstract policy goals of prohibition or legalization), harm reduction is pragmatic. Harm reduction takes a pragmatic stance about risk behaviors. One aspect of this pragmatic orientation is the acceptance that individuals will engage in the activity regardless of legal prohibition, especially when eradication of the activity is unrealistic if not impossible. Thus, even if a country criminalizes the behaviors of SW and MSM, harm reduction practices focus on HIV prevention and treatment. The objective is thus to reduce the harm associated with the activity. Indeed, the neutrality principle (outlined above) and the pragmatic principle of harm reduction are related. By assessing law in pragmatic terms, harm reduction need not engage with the normative commitments underlying prohibition or decriminalization [53]. The pragmatic analysis also focuses on the harms caused by criminalization. Thus harm reductionists pay heed to the harms that arise out of the legal framework and the consequences of criminalisation, such as exclusion from health services, or health and social impact of for example, imprisonment.

## Recommendations

In line with global arguments on this subject, and drawing on prevailing human rights discourses and pedagogy, the paper offers two approaches to HIV response in Ghana for MSM and SW - abolitionism and instrumentalism. The abolitionist approach reflects the UN Human Rights Council call of 2009 for States to eliminate laws that are counterproductive to HIV prevention, treatment and care, argue for complete and immediate repeal of all rights-limiting laws including sections of the Criminal Code of the 1960 that deny key populations their rights. The authors endorse this approach as most protective of the human rights of key populations. In line with the abolitionist approach, attempts at instituting measures to remove punitive laws, policies, practices, that block effective responses to AIDS have been made [11]. They have not been successful.

We detail below the promise of upholding rights (in the short-term or long-term) through the alternative strategy of instrumentalism. The instrumentalist approach recognizes that legal codes won’t be repealed overnight, and allows for flexibility for public health services to be delivered to persons engaged in legally outlawed activities, as doing so will ultimately impact positively on the health of the general population. The recent pilot of “Drop in Centers” (DICs) in Ghana may be a sign that Ghana is gradually buying into the instrumentalist approach – fusing human rights and harm reduction into the national response to HIV. DICs are stigma-free spaces where key populations (in the case of Ghana FSWs and MSM) can access basic health care services; HIV counseling and testing; STI screening and treatment; family planning information; and some contraceptive methods. While a DIC manager with a clinical background is responsible for the provision of health care services and the facility’s routine administrative tasks, the manager is supported by outreach workers, who are predominantly FSWs. Outreach workers create demand for the center’s services and encourage their peers to attend. The Ghana AIDS Commission recently indirectly collaborated with various non-state actors to pilot peer-educators’ led DICs and key populations-friendly clinics. These aimed to link key populations to the continuum of care for HIV-related services. A recent evaluation of Ghana’s DIC pilot project reveal that DICs promote such community engagement, providing safe, convenient spaces for socializing and discussion of issues as well as provision of health services [54]. One key factor in the success of DICs as a service delivery model has been the meaningful engagement of the key populations they serve. DICs, as outlined, are implemented with the acquiescence of state actors such as the Ghana AIDS Commission, take an instrumentalist approach that directly targets the provision of health services. As outlined earlier, the Ghana AIDS Commission is the only national coordinating agency of HIV actions, backed by law. While recognizing the value of addressing HIV-related service needs of key populations, the current legal environment precludes governmental agencies direct involvement in such interventions. Thus non-governmental organizations are encouraged and supported to provide such services.

Other instrumentalist approaches work to protect or empower key populations in general, which may alleviate the stigma and discrimination that, as shown above, can deter key populations from seeking health care. For example, Williamson et al. [9] present a conceptual framework for an HIV and key populations-related discrimination reporting system in Ghana. Aimed at facilitating access to justice in Ghana, a web-based discrimination reporting system, which links the Commission on Human Rights and Administrative Justice (CHRAJ) to civil society organizations through case reporting, follow-up, and other mechanisms that link people living with HIV and key populations to legal services. In 2016, data from this reporting system show that 21 of 78 reports by PLHIV and key populations to the Commission for Human Rights and Administrative Justice (CHRAJ) were complaints about disclosure of confidential health information compared to three reports of denial of health services. Unfortunately, the CHRAJ report does not provide additional context. To obtain further insights, assessments of human rights issues as well as quality of services for key populations are recommended.

## Conclusion

The guiding documents of Ghana’s AIDS response do not conceal their support for key populations. Both the national strategic plan for HIV and the national policy for HIV/STI advocate that public health institutions provide public health services to key populations. Impediments to key populations’ access to HIV prevention and care services are acknowledged by the guiding documents, and yet the right to the highest attainable standard of health, without qualification, is a fundamental human right. The implications of such rights curtailment are discussed. The paper then presents two approaches to addressing the problem – abolitionist and instrumentalist approaches. Either approach, at minimum, calls on Ghana to remove impediments to public health services provision to these populations. While it centrally endorses an abolitionist approach, as an interim measure, this paper makes a moderate call to the Ghana government to support these instrumentalist efforts to promote the delivery of public health services to key populations. Even without legitimizing their status and actions in the legal codes, this may be a start to protecting their rights and achieving public health goals.

## Abbreviations

DICs: Drop in Centers
FSW: Female sex workers
HIV: Human Immunodeficiency Virus
IBBSS: Integrated Biological and Behavioral Surveillance Survey
ICCPR: International Covenant on Civil and Political Rights
ICESCR: International Covenant on Economic, Social and Cultural Rights
MSM: Men who have sex with men
NGOs: Non-governmental organizations
NSP: National strategic plan
PWID: Persons who inject drugs
SWs: Sex workers
TAC: Treatment Action Campaign
UDHR: Universal Declaration of Human Rights
UN: United Nations
UNAIDS: Joint United Nations Programme on HIV/AIDS
UNESCO: United Nations Educational, Scientific, and Cultural Organization
WHO: The World Health Organization

## Footnotes

1 The UNAIDS Terminology Guidelines (2011) advises against the use of most at risk populations, as it is viewed as stigmatizing. It advocates for the use of “key populations” (both key to the epidemic’s dynamics and key to the response), instead.

2 The WHO definition for a generalized epidemic is when the prevalence is 1% or greater in the general population

3 Where unnatural carnal knowledge means sexual intercourse with a person in an unnatural manner or with an animal.

## References

[1] Kass NE (2005). An ethics framework for public health and avian influenza pandemic preparedness. Yale J Biol Med.

[2] Baum NM, Gollust SE, Goold SD, Jacobson PD (2007). Looking ahead: addressing ethical challenges in public health practice. J Law Med Ethics.

[3] Daniels N, Bryant J, Castano RA, Dantes OG, Khan KS, Pannarunothai S (2000). Benchmarks of fairness for health care reform: a policy tool for developing countries. Bull World Health Organ.

[4] Gostin L, Mann JM (1994). Towards the development of a human rights impact assessment for the formulation and evaluation of public health policies. Health Human Rights.

[5] ten Have M, de Beaufort ID, Mackenbach JP, van der Heide A (2010). An overview of ethical frameworks in public health: can they be supportive in the evaluation of programs to prevent overweight?. BMC Public Health.

[6] World Health Organization. Constitution of the World Health Organization, as adopted by the International Health Conference, New York, 19–22 June 1946; signed on 22 July 1946 by the representatives of 61 States (Official Records of the World Health Organization, no. 2, p. 100) and entered into force on 7 April 1948. WHO, Geneva, Switzerland. 1948. Available at http://apps.who.int/iris/bitstream/10665/121457/1/em_rc42_cwho_en.pdf.

[7] Overs C, Hawkins K (2011). Can rights stop the wrongs? Exploring the connections between framings of sex workers’ rights and sexual and reproductive health. BMC Int Health Hum Rights.

[8] Persson A, Ellard J, Newman C, Holt M, de Wit J (2011). Human rights and universal access for men who have sex with men and people who inject drugs: a qualitative analysis of the 2010 UNGASS narrative country progress reports. Soc Sci Med (1982).

[9] Williamson RT, Wondergem P, Amenyah R (2014). Using a reporting system to protect the human rights of people living with HIV and key populations: a conceptual framework. Health Hum Rights.

[10] Joint United Nations Programme on HIV/AIDS (UNAIDS): Guidance Note on HIV and Sex Work. UNAIDS. Geneva, Switzerland; 2009. Available at http://www.unaids.org/sites/default/files/sub_landing/files/JC2306_UNAIDS-guidance-note-HIV-sexwork_en.pdf.

[11] UNAIDS Ghana (2010). A report submitted by the united nations programme on HIV/AIDS (UNAIDS) to the office of the Human Rights Council on the universal periodic review.

[12] Solace Brothers Foundation, The Initiative for Equal Rights, Center for International Human Rights of Northwestern University School of Law, Heartland Alliance for Human Needs & Human Rights, Global Initiative for Sexuality and Human Rights (2015). Human rights violations against Lesbian, Gay, Bisexual, and Transgender (LGBT) people in Ghana: a shadow report submitted for consideration at the 115th Session of the Human Rights Committee October 2015, Geneva.

[13] Government of Ghana (1960). The criminal code of the Republic of Ghana, 1960 (Act 29).

[14] Duvall S, Sanon P, M. Maeda M, Daniel U (2015). HPP key populations policy analysis: countries along the Abidjan-Lagos Corridor (Côte d’Ivoire, Ghana, Togo, Benin, and Nigeria) and Burkina Faso.

[15] UNDP HAG (2012). Global commission on HIV and the law: risk, rights and health.

[16] Poteat T, Diouf D, Drame FM, Ndaw M, Traore C, Dhaliwal M, Beyrer C, Baral S (2011). HIV risk among MSM in Senegal: a qualitative rapid assessment of the impact of enforcing laws that criminalize same sex practices. PLoS One.

[17] Malone B (2011). Uganda's “Kill The Gays” Bill shelved again. Reuters; May 13 2011.

[18] Obadare E: Sexual struggles and democracy dividends. In: Contesting the Nigerian State. Palgrave Macmillan US, Springer; 2013: 199-215. Available at https://link.springer.com/chapter/10.1057/9781137324535_8.

[19] Meier BM, Gelpi A, Kavanagh MM, Forman L, Amon JJ (2015). Employing human rights frameworks to realize access to an HIV cure. J Int AIDS Soc.

[20] Ghana Statistical Service (GSS), Ghana Health Service (GHS), & ICF Macro. The 2014 Ghana Demographic and Health Survey. Ghana Statistical Service, Accra, Ghana; 2015. Report is available at https://dhsprogram.com/pubs/pdf/FR307/FR307.pdf.

[21] Eba PM (2015). HIV-specific legislation in sub-Saharan Africa: a comprehensive human rights analysis. African Human Rights Law J.

[22] Boggio A, Zignol M, Jaramillo E, Nunn P, Pinet G, Raviglione M (2008). Limitations on human rights: are they justifiable to reduce the burden of TB in the era of MDR- and XDR-TB?. Health Human Rights.

[23] Nations U (1948). UN general assembly, universal declaration of human rights, 10 December 1948, 217 A (III).

[24] United Nations General Assembly. International Covenant on Civil and Political Rights. Adopted and opened for signature, ratification and accession by General Assembly resolution 2200A (XXI) of 16 December 1966. Available at http://www.ohchr.org/Documents/ProfessionalInterest/ccpr.pdf.

[25] United Nations General Assembly: International Covenant on Economic, social and Cultural Rights, United Nations, treaty series, 993(3). United Nations, New York, NY; USA. 1966.

[26] London L, Orner PJ, Myer L (2008). ‘Even if you’re positive, you still have rights because you are a person’: human rights and the reproductive choice of HIV-positive persons. Dev World Bioeth.

[27] AaHRRU UNDP (2008). Compendium of key documents relating to human rights and HIV in Eastern and Southern Africa.

[28] Jürgens R, Csete J, Amon JJ, Baral S, Beyrer C (2010). People who use drugs, HIV, and human rights. Lancet.

[29] Amon JJ, Baral SD, Beyrer C, Kass N (2012). Human rights research and ethics review: protecting individuals or protecting the state?. PLoS Med.

[30] Ghana AIDS Commission: Ghana Country AIDS Progress Report. Reporting period January 2010 – December 2011. March 2012. Ghana AIDS Commission, Accra, Ghana. Available at http://files.unaids.org/en/dataanalysis/knowyourresponse/countryprogressreports/2012countries/ce_GH_Narrative_Report%5B1%5D.pdf.

[31] Altman D, Aggleton P, Williams M, Kong T, Reddy V, Harrad D, Reis T, Parker R (2012). Men who have sex with men: stigma and discrimination. Lancet.

[32] Thoreson R, Cook S: Thoreson R, Cook S. Nowhere to turn: blackmail and extortion of LGBT people in sub-Saharan Africa. International Gay and Lesbian Human Rights Commission (IGLHRC), Johannesburg; 2011. Available at http://www.outrightinternational.org/sites/default/files/484-1.pdf.

[33] Djomand G, Quaye S, Sullivan PS (2014). HIV epidemic among key populations in west Africa. Curr Opin HIV AIDS.

[34] Laurent C, Seck K, Coumba N, Kane T, Samb N, Wade A, Liégeois F, Mboup S, Ndoye I, Delaporte E (2003). Prevalence of HIV and other sexually transmitted infections, and risk behaviours in unregistered sex workers in Dakar, Senegal. AIDS.

[35] Beyrer C. Hidden yet happening: the epidemics of sexually transmitted infections and HIV among men who have sex with men in developing countries. Sexually transmitted infections. 2008;84(6):410-12. 10.1136/sti.2008.033290 19028936

[36] Millett GA, Jeffries WL, Peterson JL, Malebranche DJ, Lane T, Flores SA, Fenton KA, Wilson PA, Steiner R, Heilig CM (2012). Common roots: a contextual review of HIV epidemics in black men who have sex with men across the African diaspora. Lancet.

[37] UNAIDS (2012). UNAIDS, UNAIDS guidance note: key programmes to reduce stigma and discrimination and increase access to justice in national HIV responses (Geneva, Switzerland: UNAIDS, 2012).

[38] Ghana Ro (1996). The Constitution of the Republic of Ghana (AMENDMENT) ACT, 1996. Date of ASSENT: 16th December 1996.

[39] Ghana AIDS Commission, Ministry of Health, NACP: Republic of Ghana National HIV and, STI Policy. Ghana AIDS Commission, Accra, Ghana; 2013. Available at http://www.ghanaids.gov.gh/gac1/pubs/Ghana%20National%20HIV%20and%20AIDS%20STI%20Policy.pdf.

[40] Multimedia Group Ghana (2016). Multimedia Group Online news item, dated July 5 2016.

[41] Newspaper T (2016). Online news item of the today newspaper, dated March 1 2016.

[42] Citi FM Ghana (2016). Citi FM online news item, dated February 29 2016.

[43] Lowndes CM, Alary M, Belleau M, Bosu WK, Kintin DF, Nnorom JA, Seck, K, Victor-Ahuchogu J, and Wilson D. West Africa HIV/AIDS Epidemiology and Response Synthesis. Characterisation of the HIV epidemic and response in West Africa: Implications for prevention. World Bank, Global AIDS Monitoring and Evaluation Team (GAMET): ACT Africa, November 2008. Available at http://siteresources.worldbank.org/INTHIVAIDS/Resources/375798-1132695455908/WestAfricaSynthesisNov26.pdf.

[44] Robins S (2006). “Rights” to “Ritual”: AIDS activism in South Africa. Am Anthropol.

[45] UNAIDS, WHO: UNGASS declaration of commitment on HIV/AIDS. UNAIDS, Geneva, Switzerland. Geneva, Switzerland; 2001.

[46] Joint United Nations Programme on HIV/AIDS (UNAIDS): Joint Action for Results UNAIDS Outcome Framework 2009–2011. WHO, Geneva, Switzerland; 2009. Available at http://awdflibrary.org/bitstream/123456789/560/1/jc1713_joint_action_en.pdf.

[47] UNESCO. Review of Homophobic Bullying in Educational Institutions Prepared for the International Consultation on Homophobic Bullying in Educational Institutions Rio de Janeiro, Brazil, 6-9 December 2011 . UNESCO, Paris, France. Available at http://unesdoc.unesco.org/images/0021/002157/215708e.pdf.

[48] Cohen J, Wolfe D (2008). Harm reduction and human rights: finding common cause. AIDS.

[49] Gruskin S, Cottingham J, Hilber AM, Kismodi E, Lincetto O, Roseman MJ (2008). Using human rights to improve maternal and neonatal health: history, connections and a proposed practical approach. Bull World Health Organ.

[50] Erdman JN (2011). Access to information on safe abortion: a harm reduction and human rights approach. Harvard J Law Gender.

[51] Wade AS, Larmarange J, Diop AK, Diop O, Gueye K, Marra A, Sene A, Enel C, Niang Diallo P, Toure Kane NC (2010). Reduction in risk-taking behaviors among MSM in Senegal between 2004 and 2007 and prevalence of HIV and other STIs. ELIHoS Project, ANRS 12139. AIDS Care.

[52] MOH (2011). Results from the 2011 HIV/STI Integrated Biological and Behavioral Surveillance (IBBS) in Ghana.

[53] Hathaway AD (2001). Shortcomings of harm reduction: toward a morally invested drug reform strategy. Int J Drug Policy.

[54] Bloom S, Cannon A, Negroustoueva S, Okonofua F, Wong L, Nakigozi G, Atuyambe L, Kamya M, Makumbi F, Chang L: A performance evaluation of the National HIV Prevention Program for FSW and MSM in Ghana. Afr J Reprod Health 2013;17(4 Spec No):9–16.

